# Neuroimmune and Mu-Opioid Receptor Alterations in the Mesocorticolimbic System in a Sex-Dependent Inflammatory Pain-Induced Alcohol Relapse-Like Rat Model

**DOI:** 10.3389/fimmu.2021.689453

**Published:** 2021-09-20

**Authors:** Javier Cuitavi, Jesús David Lorente, Yolanda Campos-Jurado, Ana Polache, Lucía Hipólito

**Affiliations:** Department of Pharmacy and Pharmaceutical Technology and Parasitology, University of Valencia, Burjassot, Spain

**Keywords:** mu-opioid receptor, alcohol, pain, alcohol deprivation effect, microglia, neuroinflammation

## Abstract

Evidence concerning the role of alcohol-induced neuroinflammation in alcohol intake and relapse has increased in the last few years. It is also proven that mu-opioid receptors (MORs) mediate the reinforcing properties of alcohol and, interestingly, previous research suggests that neuroinflammation and MORs could be related. Our objective is to study neuroinflammatory states and microglial activation, together with adaptations on MOR expression in the mesocorticolimbic system (MCLS) during the abstinence and relapse phases. To do so, we have used a sex-dependent rat model of complete Freund’s adjuvant (CFA)-induced alcohol deprivation effect (ADE). Firstly, our results confirm that only CFA-treated female rats, the only experimental group that showed relapse-like behavior, exhibited specific alterations in the expression of phosphorylated NFκB, iNOS, and COX2 in the PFC and VTA. More interestingly, the analysis of the IBA1 expression revealed a decrease of the microglial activation in PFC during abstinence and an increase of its expression in the relapse phase, together with an augmentation of this activation in the NAc in both phases that only occur in female CFA-treated rats. Additionally, the expression of IL1β also evidenced these dynamic changes through these two phases following similar expression patterns in both areas. Furthermore, the expression of the cytokine IL10 showed a different profile than that of IL1β, indicating anti-inflammatory processes occurring only during abstinence in the PFC of CFA-female rats but neither during the reintroduction phase in PFC nor in the NAc. These data indicate a downregulation of microglial activation and pro-inflammatory processes during abstinence in the PFC, whereas an upregulation can be observed in the NAc during abstinence that is maintained during the reintroduction phase only in CFA-female rats. Secondly, our data reveal a correlation between the alterations observed in IL1β, IBA1 levels, and MOR levels in the PFC and NAc of CFA-treated female rats. Although premature, our data suggest that neuroinflammatory processes, together with neural adaptations involving MOR, might play an important role in alcohol relapse in female rats, so further investigations are warranted.

## 1 Introduction

Chronic alcohol intake is the third cause of death in developed countries (1, 2), and it is related to many medical conditions, since it is one of the most harmful drugs (3). Alcohol use disorder (AUD) is a recurrent pathological condition that is characterized by repeated relapse episodes after periods of prolonged abstinence (4). Nowadays, pharmacotherapeutic strategies to prevent alcohol relapse have not always shown a great rate of success, probably because there are different aspects (i.e., stress, co-occurrence of other pathologies) that can be developed during abstinence and might impact the efficacy of the anti-relapse pharmacological treatments (5–7). This point is crucial because a better knowledge of neurochemical adaptations occurring during the abstinence and the relapse phases in the presence of different factors (i.e., gender, genetic, contextual, environmental) might help us to develop better therapeutical strategies tailored to the characteristics of the patients.

Mu-opioid receptor (MOR) antagonists (naltrexone and nalmefene) are one of the selected anti-relapse treatments. These medications help reduce the risk of relapse and promote less hazardous drinking (8). Their use is based on the involvement of these receptors in the rewarding and reinforcing properties of alcohol. MORs located in different areas of the mesocorticolimbic system (MCLS) such as the ventral tegmental area (VTA), the nucleus accumbens (NAc), and the prefrontal cortex (PFC) are activated by alcohol-induced endorphin release (9) or alcohol metabolism derivatives (10) to indirectly increase dopaminergic neurons activity and, finally, drive some of the behavioral consequences of alcohol administration. Very interestingly, a variety of literature has revealed a relationship between MOR activation and neuroinflammatory events. On the one hand, MORs on microglia seem to enhance the release of neuroinflammatory mediators, cytokines, and chemokines after their activation (11, 12). On the other hand, pro-inflammatory cytokines such as IL1β, IL6, and TNFα can regulate MOR expression on some immune cells and neurons (13–16). Although this relationship is still not fully understood, cross talk between Toll-like receptor 4 (TLR4) and MORs at the intracellular level seems to participate, as has been recently explained in a revision (17, 18).

Alcohol has also shown to trigger neuroinflammatory events through the TLR4 pathway. In fact, Guerri’s laboratory has shown in the last decade that alcohol intermittent administration induces proinflammatory cytokine release through the activation of TLR4, promoting neuronal adaptations (19–23). The reported results by this and other groups have shown that neuroinflammatory events appeared during alcohol administration and early abstinence but might also play an important role in alcohol relapse. In this sense, Ezquer and colleagues have shown very recently that the prevention of alcohol-induced oxidative stress and neuroinflammation in key brain areas of the MCLS through the intranasal administration of exosomes from human mesenquimal cells decreased alcohol intake and blunted alcohol relapse-like binge drinking in female rats bred as alcohol consumers (24).

We hypothesize that, during the abstinence and relapse phases, specific adaptations of the neuroinflammatory state and changes in MOR expression can be developed in selected brain areas of the MCLS of the rats showing relapse-like behavior. To further investigate this hypothesis, we selected a sex-dependent inflammatory pain-induced alcohol deprivation effect (ADE) rat model developed recently by our laboratory based on the complete Freund’s adjuvant (CFA) (25). In this model, inflammatory pain could act as a risk factor toward alcohol relapse only in female rats, which were the only group that manifested the ADE (25). It is interesting to note that other animal models to investigate the relapse phenomenon induce alcohol-relapse-like behavior *per se* (i.e., four-bottle choice ADE paradigm), making the investigation of the biochemical adaptation occurring during abstinence in relapsing and non-relapsing individuals difficult. The use of the model proposed here allows us to do this since males and control females do not develop ADE, and the only group showing a significant increase of alcohol intake is the female rats suffering from inflammatory pain. Our objective here, by using this model, is to explore neuroinflammation (measuring phosphorylated NFkB, iNOS, COX2 expression), the activation of microglia (through the expression of IBA1), and cytokine (IL1β and IL10) expression in the abstinence and the reintroduction relapse phases in males and females with or without inflammatory pain in parallel to the expression of the MORs in selected areas of the MCLS.

## 2 Methods

### 2.1 Animals

One hundred fourteen Sprague Dawley adult rats, females and males, were used (Envigo^®^, Barcelona, Spain). All the animals were kept in inverted light/dark (12/12 h, light on at 22:00) controlled cycles, temperature 23 ± 1°C, and 60% humidity. Each animal was individually housed in a standard plastic cage (42 × 27 × 18 cm^3^) with food and tap water provided *ad libitum* throughout the experimental period. Rats were housed in the animal facilities of the University of Valencia. Animal protocols followed in this work were approved by the Animal Care Committee of University of Valencia and were strictly adhered to in compliance with the EEC Council Directive 63/2010 and Spanish laws (RD 53/2013).

### 2.2 Ethanol Intermittent Access Model and Pain Induction

**Figure 1:**
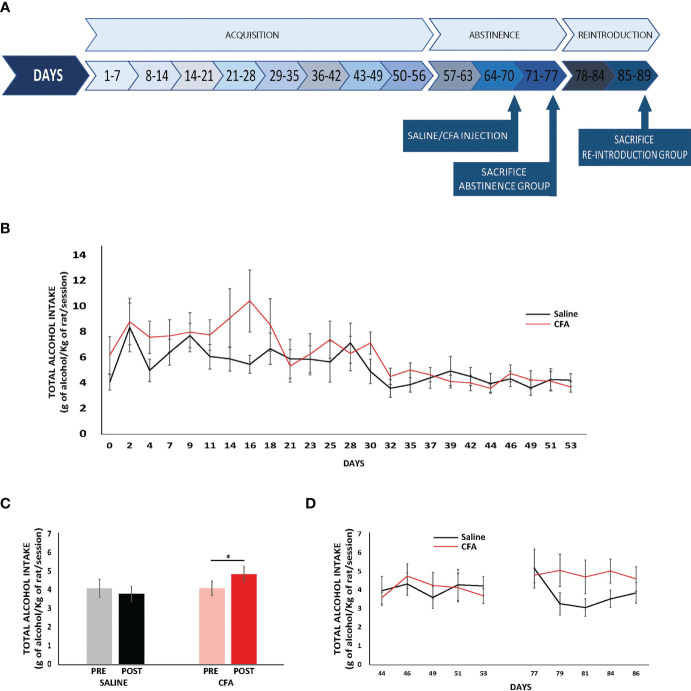
Inflammatory pain induces alcohol relapse in female rats. **(A)** Schematic of the alcohol and inflammatory pain experimental design. **(B)** Alcohol intake during the acquisition period. Data are expressed as mean ± SEM of each consumption day (n = 10–11/group) (ANOVA for repeated measures, p > 0.05). **(C)** Mean ± SEM (left saline-female and right CFA-female) of total alcohol intake (g/kg/day) of the 5-day pre- (basal, lighter bar) and post-abstinence (darker bars) shown in gray/black for the saline-treated group (n = 10/group) and in pink/red for the CFA-treated group (n = 11/group) (ANOVA for repeated measures followed by Bonferroni multiple comparisons, *p < 0.05). **(D)** Mean ± SEM of total alcohol intake (g/kg/day) of the last 5 consumption days pre-abstinence and the 5 consumption days post-abstinence (n = 10–11/group); saline-females in black and CFA-females in red (ANOVA for repeated measures, p > 0.05).

In this procedure, 40 males and 52 females (total n = 92 rats) followed the classical ethanol intermittent access (IA) model (26) shown in **Figure 1A** in combination with a CFA-based inflammatory pain model (27–29), as we have previously described (25). Rats had free access to 20% ethanol solution and water on Monday, Wednesday, and Friday during 24 h for 8 weeks. After this acquisition period, alcohol was removed for 3 weeks to force a period of abstinence. On the first day of the third week of abstinence, animals received 0.1 ml of CFA (Calbiochem), or sterile saline, in the plantar surface of the hindpaw. The intraplantar injection was made alternately in the right or left hindpaw of the animals in a counterbalance fashion. At the end of 3 weeks of forced abstinence, alcohol was reintroduced following the same IA procedure for five more sessions. Twenty-four hours after the last alcohol session, animals were sacrificed by either isoflurane when the brain was freshly removed or pentobarbital overdose when animals were perfused with paraformaldehyde. Rats belonging to the abstinence groups were sacrificed in the same way after completing 3 weeks of abstinence, on the day when alcohol was supposed to be reintroduced. It is important to notice that brain tissue obtained from all males and 31 females following this protocol were obtained from rats used in a previous study (25). This decision was taken to reduce the number of animals used in this study in compliance with the 3Rs and animal care regulations. Nonetheless, to prove the reproducibility in the animal model first described by Lorente and collaborators (25), a new batch of 21 females was run. Therefore, we only include the alcohol consumption data from these females on this paper (see *Inflammatory Pain Induces Alcohol Relapse in Females* and **Figure 1**). The alcohol consumption data from the males and from the rest of the females can be found in Lorente et al. (2021) (25).

In addition to the rats that followed IA, a control group for the semiquantitative techniques (Western blot and immunofluorescence) composed of 10 females and 12 males (n = 22), only having access to water, was run at the same time. Half of the animals from this group were sacrificed by isoflurane when the brain was freshly removed whereas the other half were sacrificed by pentobarbital overdose when animals were perfused with paraformaldehyde.

Therefore, 10 experimental groups were organized by sex:

1. Male (n = 52): control group (rats that had access only to water) and four groups that followed the IA protocol, SAL-A (rats without pain, sacrificed during abstinence), SAL-R (rats without pain sacrificed after reintroduction), CFA-A (rats in pain sacrificed during abstinence), and CFA-R (rats in pain sacrificed after reintroduction);
2. Female (n = 62): control group and four groups that followed the IA protocol, SAL-A, SAL-R, CFA-A, and CFA-R.

### 2.3 Western Blot

This technique was used to measure the expression levels of phosphorylated-NFκB, iNOS, COX2, IL1β, IL10, and MOR, in different brain areas from control and IA animals sacrificed by isoflurane overdose. Freshly removed brains from 30 females (n = 6/condition) and 30 males (n = 6/condition) were immediately frozen in dry ice and stored at -80°C until the Western blot experiment was performed. Then, PFC, NAc, and VTA were dissected in both hemispheres, and the tissues were homogenized in cold lysis buffer (1% IGEPAL CA-630, 20 mM Tris–HCl pH 8, 130 mM NaCl, 10 mM NaF, and 1% protease inhibitor cocktail, Sigma, St. Louis), using 0.5 ml of lysis buffer each 250 mg of tissue. The homogenate extracts were kept in ice for 30 min. Afterward, samples were immediately centrifuged at 15,000 g for 15 min at 4°C; the supernatant was collected to determine the protein concentration by using a Bradford protein assay kit (Bio-Rad). This procedure was adapted from one previously used (30, 31).

Western blot was used to determine the expression levels of the abovementioned proteins in the homogenate extracts. To do so, we followed a previously used protocol described by Lorente and collaborators (25). The following primary antibodies were used: rabbit IgG anti-MOR (1:1000, Abcam ab134054) (32), rabbit IgG anti-phosphorylated-NFκB p65 (1:1000, Abcam ab86299) (33), rabbit IgG anti-iNOS (1:500, Abcam ab204017) (34), rabbit IgG anti-COX2 (1:1000, Abcam ab52237) (35), rabbit IgG anti-IL1β (1:2500, Invitrogen PA5-79485), and rabbit anti-IL10 (1:2500, Abcam ab9969) (36). Goat IgG anti-rabbit (1:1000, Bio-Rad 1706515) was used as a secondary antibody. Mouse IgG anti-GAPDH conjugated with HRP (1:1000, Invitrogen MA5-15738-HRP) (37) was used to detect GAPDH as a protein loading control. When the membranes were incubated with more than one primary antibody, before probing with the second or third antibody, they were treated with Restore™ Western Blot Stripping Buffer (Thermo Fisher) for 15 min. Finally, the intensity of the bands was expressed as arbitrary units and normalized to GAPDH band intensity. Relative protein levels to control were determined by setting the control group to 100% and calculating the respective percentages for each band. All samples (20 µg) were run in duplicate, obtaining an average of the % from control for each sample. A representative image obtained from each group included in the Western blot analysis is shown on **Supplementary Figure 1**.

### 2.4 Immunofluorescence

Microglial activation was assessed by measuring ionized calcium-binding adapter molecule 1 (IBA1) expression with an immunofluorescence assay (38). To do so, control and IA animals were used. Thirty-two females (control: n = 4; SAL-A, SAL-R, CFA-A, CFA_R: n = 7/condition) and 22 males (control: n = 6; SAL-A, SAL-R, CFA-A, CFA-R: n = 4/condition) were anesthetized by injecting pentobarbital and followed a procedure of cardiac perfusion with 200 ml paraformaldehyde 0.4% in phosphate buffer (PB) 0.1 M. Brains were extracted and kept in the same perfusion solution for 20 h at 4™C. After that, they were transferred to sucrose 30% in PB 0.1 M for 3 days. Following this, 40-µm brain slices were obtained in four series on a freezing microtome and were stored at -80°C in sucrose 30% in PB 0.1 M until their use. Immunofluorescence was performed as previously described (25). The rabbit IgG anti-IBA1 (1:2000, Wako 019-19741) primary antibody (39) and the donkey IgG anti-rabbit Alexa Fluor^®^ 488 (1:1000, Invitrogen A32790) secondary antibody were used.

Images from PFC, NAc, and VTA were obtained with a ×20 objective (Leica Biosystems, Germany; images size 441 × 330 µm). We obtained six to eight images (from both hemispheres) per area, and mean gray intensity (MGI) was analyzed by means of FIJI software. Results were expressed in percentage of the control group. A representative microphotography of the DAPI and IBA1 staining is shown in **Supplementary Figures 1** and **2**.

### 2.5 Statistical Analysis

Results are shown as mean ± standard error of the mean (SEM). To perform the statistical analysis, the 26.0 version of the SPSS program was used. The Kolmogorov–Smirnov test and Levene’s test were performed to assess the normality and the homogeneity of the variance of the data. When an experimental variable (i.e., alcohol consumption) was continuously measured (i.e., along the experimental procedure), an ANOVA for repeated measures was applied followed by Bonferroni multiple comparisons when appropriate. For Western blot and immunofluorescence, the control group (non-treated rats not exposed to alcohol) was used to allow us to compare the rest of the groups by normalizing them to control in a percentage. For these experiments, the two-way ANOVA test was performed followed by Bonferroni multiple comparisons when significant differences in the main effects (pain; abstinence) or in the interaction were detected. In all the statistical tests, a 95% confidence level was set.

## 3 Results

### 3.1 Inflammatory Pain Induces Alcohol Relapse in Female Rats

**Figure 1B** shows the total alcohol intake rate (g/kg/session) of the new batch of 21 females run in this study along the days of the acquisition period. Very importantly, no significant differences were found in alcohol consumption before the forced abstinence period between females that were afterward injected with CFA and the ones injected with saline (**Figure 1B**; ANOVA repeated measures, F(1,19) = 1.048 p = 0.319). **Figure 1C** shows the averages of the alcohol intake levels during the last 5 days of acquisition and the 5 days of reintroduction. Interestingly, the repeated-measure ANOVA showed significant differences (F(1,19) = 4.615 p = 0.045). In fact, the *post-hoc* test revealed that CFA-female rats significantly increased their consumption levels regarding the basal levels whereas animals injected with saline did not change their consumption levels (**Figure 1D** shows single-day data from the last 5 days of acquisition and the 5 days of reintroduction. Repeated-measure ANOVA showed that there are no differences in the ethanol intake between saline and CFA female rats through time (F(1,19) = 0.858 p = 0.366) but, as can be observed in the figure, CFA female rats presented higher levels of ethanol consumption than saline ones after reintroduction every testing day.

### 3.2 Biochemical Analysis of Neuroinflammatory Mediators, IBA-1, IL1β, IL10, and MOR Expression in PFC, NAc, and VTA of Saline and CFA-Treated Male and Female Rats in Abstinence and Reintroduction Phases

Statistical analysis and values of the F and p for the main effects *pain* and *abstinence/re-introduction* and the interaction are summarized in the table of the **Supplementary Material**.

#### 3.2.1 Alcohol-Induced Neuroinflammation in PFC, NAc, and VTA of CFA-Treated Females Present Specific Alterations During Abstinence and Reintroduction Phases

As has previously been shown in the literature, during chronic alcohol administration and early abstinence (24 h), neuroinflammatory pathway is activated. Thereby, the levels of transcriptional factors (as pNFκB) and neuroinflammatory mediators (such as iNOS and COX2) are increased (20, 40). To investigate the neuroinflammatory pathway activation in NAc, PFC, and VTA of saline and CFA rats during abstinence and relapse, we measured by Western blot the levels of phosphorylated NFκB, iNOS, and COX2.

**Figure 2:**
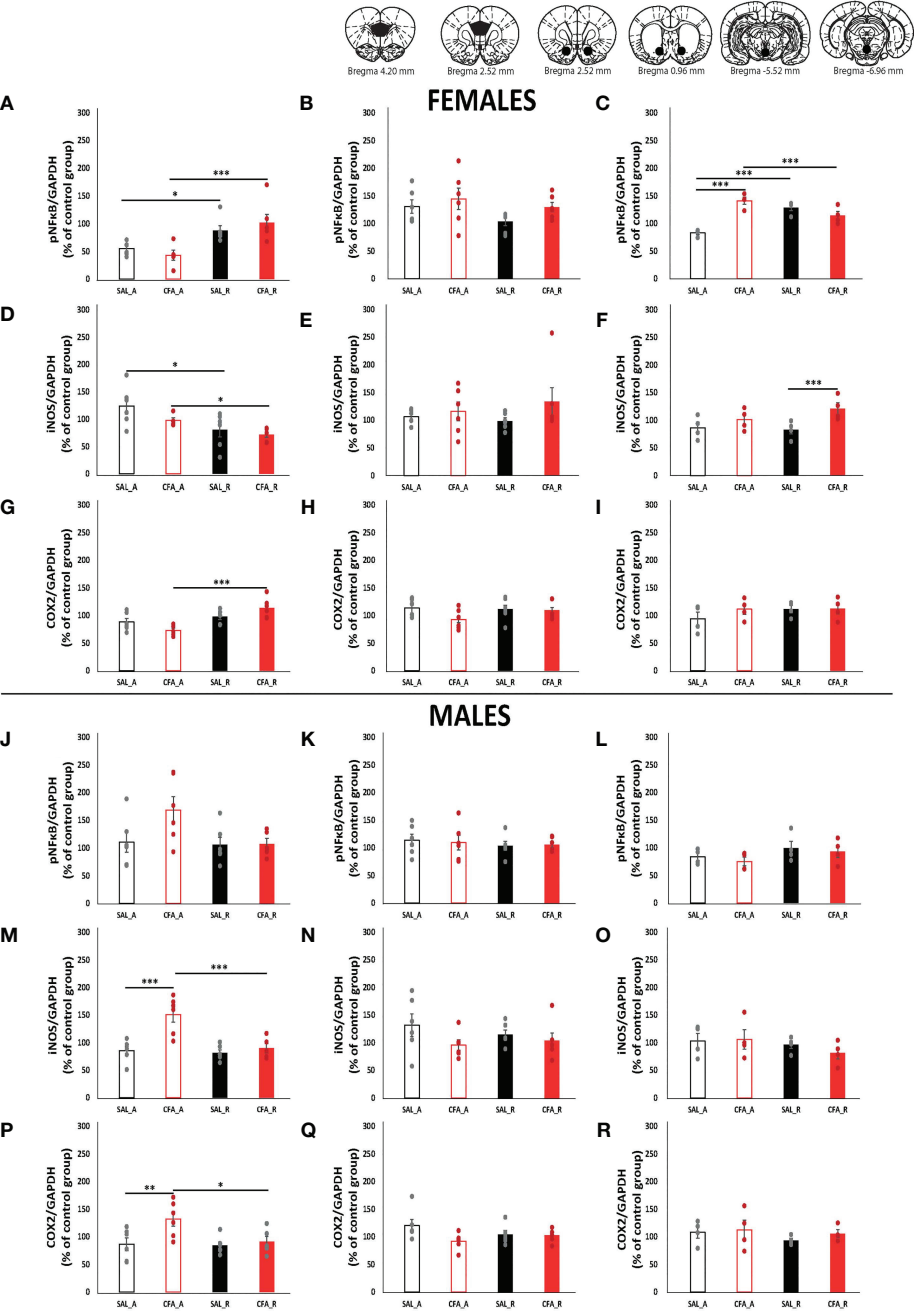
Alterations of inflammatory mediators during the abstinence and the alcohol reintroduction periods in the presence or absence of inflammatory pain in female and male rats. Data are expressed as mean ± SEM of protein levels in % relative to control (n = 4–6/group). Black and red bars represent saline and CFA-females, respectively, during abstinence (empty bars) and after abstinence (filled bars) periods. Points represent the individual data from each animal from the group. On top, schematic representations of the punched brain areas harvested to perform the Western blots. Graphs **(A–I)** gather the protein analysis of female rats and graphs **(J–R)** those from male rats; PFC, NAc, and VTA protein analyses are represented in the following order, PFC: **(A**, **D**, **G**, **J**, **M**, **P)**; NAc: **(B**, **E**, **H**, **K**, **N**, **Q)**; VTA: **(C**, **F**, **I**, **L**, **O**, **R)**. The proteins analyzed are pNFκB **(A–C**, **J–L)**, iNOS **(D–F**, **M–O)**, and COX2 **(G–I**, **P–R)**. Asterisks mark statistically significant differences in the Bonferroni multiple-comparison test applied when the two-way ANOVA detected significant differences in the main effects or in the interaction (*p < 0.05, **p < 0.01, ***p < 0.005). CFA, complete Freund adjuvant; SAL, saline; A, abstinence period; R, reintroduction period; PRC, prefrontal cortex; NAc, nucleus accumbens; VTA, ventral tegmental area; pNFκB, phosphorylated nuclear factor κB; iNOS, inducible nitric oxide synthase and COX2, cyclooxygenase 2.

The two-way ANOVA performed found statistically significant differences in main variables and/or its interaction in PFC from saline- and CFA-treated females and males. On the one hand, regarding female rats, pNFκB levels were significantly increased after alcohol reintroduction regardless of the presence of inflammatory pain (**Figure 2A**: SAL_A *vs.* SAL_R, p= 0.03; CFA_A *vs.* CFA_R, p = 0.0001). When analyzing iNOS, we observe the opposite phenomenon since its levels were significantly decreased after alcohol reintroduction, regardless of the presence of inflammatory pain (**Figure 2D**: SAL_A *vs.* SAL_R, p = 0.014; CFA_A *vs.* CFA_R, p = 0.036). Very interestingly, COX2 was the only neuroinflammatory mediator that significantly increased after reintroduction only in CFA-females, indicating a specific change derived from the development of pain during abstinence (**Figure 2G**, CFA_A *vs.* CFA_R, p = 0.0001). On the other hand, regarding male rats, no significant differences were observed when analyzing the levels of pNFκB (**Figure 2J**). However, iNOS and COX2 show statistically significant alterations between groups. Interestingly, both proteins are altered only in CFA-males during abstinence since they have significantly higher levels of those proteins during abstinence than after reintroduction, which could be a direct consequence of the presence of inflammatory pain (**Figures 2M, P**).

We also observed significant alterations in VTA from females in our animal model when analyzing pNFκB and iNOS, but we did not observe any significant changes for COX2 (**Figure 2I**). Interestingly, inflammatory pain alters pNFκB levels in a different pattern during abstinence and after alcohol reintroduction in comparison with saline-females. Indeed, CFA-females have higher pNFκB levels than SAL-females during abstinence (p = 0.0001). Nonetheless, SAL-females increase their pNFκB levels after alcohol reintroduction (p = 0.0001), whereas CFA-females decrease them (p = 0.007) (**Figure 2C**). The two-way ANOVA also found differences for the main effect pain when analyzing iNOS expression in the VTA of female rats (**Figure 2F**). In this case, iNOS expression increased only in CFA-females after alcohol reintroduction which was only significant when compared with SAL-females (p = 0.016).

Finally, the two-way ANOVA tests performed failed to find any significant differences between groups in NAc for both females and males when analyzing pNFκB, iNOS, and COX2 (**Figure 2B**: pNFκB; **Figures 2E, N**: iNOS, **Figures 2H, Q**: COX2). Additionally, we found no significant differences when comparing groups in VTA from males (**Figures 2L, O, R**).

#### 3.2.2 IBA1 Expression Is Decreased During Abstinence and Increased After Reintroduction of the Alcohol Beverages in the PFC Whereas Opposed Alterations Are Observed in the NAc of Only CFA-Treated Female Rats

**Figure 3:**
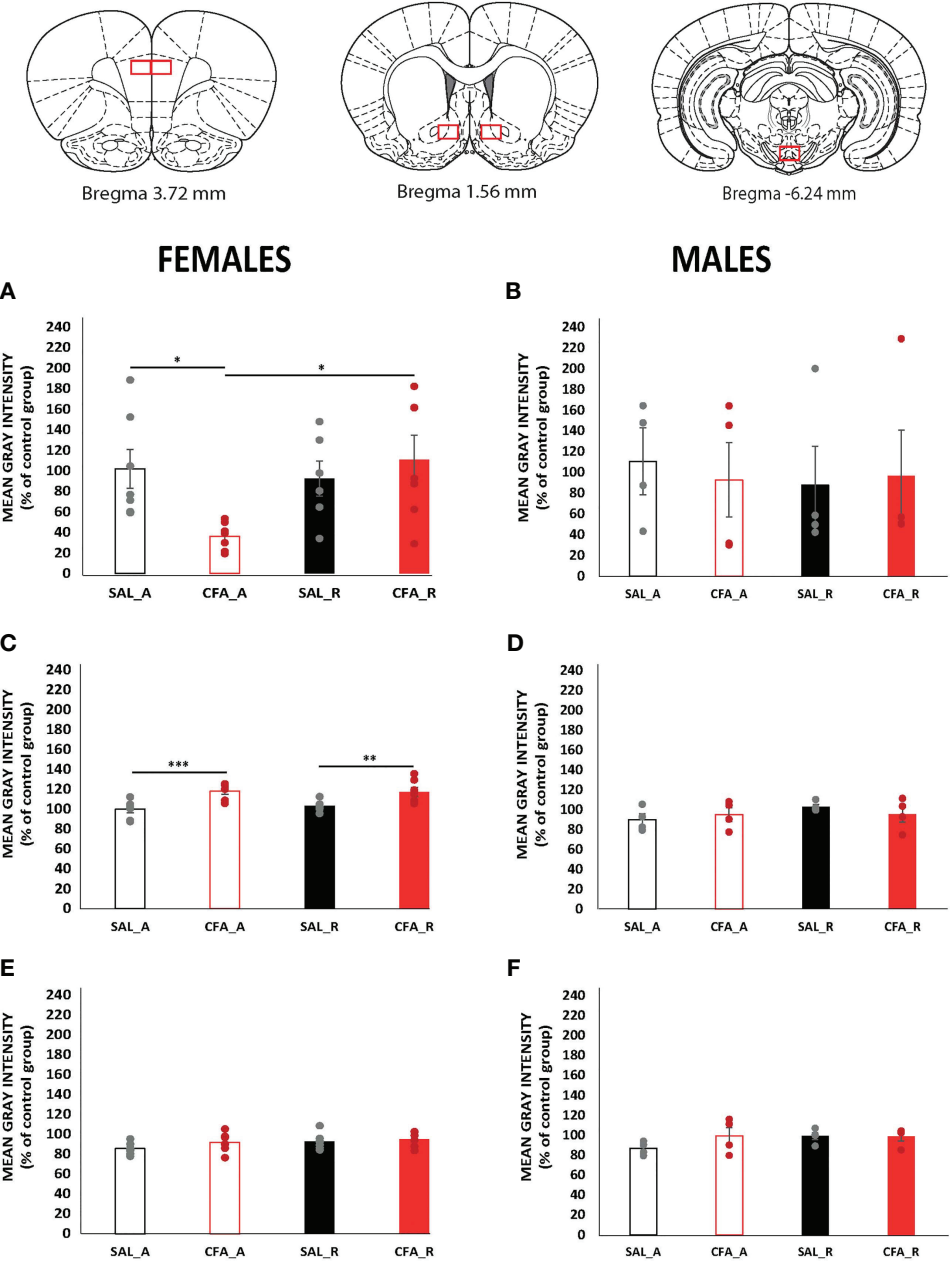
Alterations of microglial activation measured as IBA1 staining during the abstinence and the alcohol reintroduction periods in the presence or absence of inflammatory pain in female and male rats. Data are expressed as mean ± SEM of IBA1 levels in % relative to control (n = 4–7/group). Black and red bars represent saline and CFA-females, respectively, during the abstinence (empty bars) and after abstinence (filled bars) periods. Points represent the individual data from each animal from the group. On the bottom, brain schematics representing the areas where the pictures were taken (Paxinos and Watson 2006). Graphs **(A, C, E)** gather the protein analysis of female rats, and graphs **(B, D, F)** those from male rats; PFC, NAc, and VTA protein analyses are represented in the following order, PFC: **(A, B)**; NAc: **(C, D)**; VTA: **(E, F)** Asterisks mark statistically significant differences in the Bonferroni multiple-comparison test applied when the two-way ANOVA detected significant differences in the main effects or in the interaction (*p < 0.05, **p < 0.01, ***p < 0.005). CFA, complete Freund adjuvant; SAL, saline; A, abstinence period; R, reintroduction period; PFC, prefrontal cortex; NAc, nucleus accumbens; VTA, ventral tegmental area; IBA1, ionized calcium-binding adapter molecule 1.

Neuroinflammatory processes within the brain are partially regulated by glial cells. Microglia are the immune cells per excellence in the brain. Therefore, we assessed microglial activation with IBA1 immunofluorescence in brain areas of the mesocorticolimbic system, as shown in **Figure 3**.

The two-way ANOVA tests performed showed significant differences when comparing groups in female rats for PFC and NAc, but not for VTA IBA1 staining (**Figure 3E**). Regarding PFC, CFA-females show significantly lower levels during abstinence than after reintroduction (p = 0.004) and when compared to SAL-females (p = 0.009) during the same period (**Figure 3A**). Interestingly, alterations produced by pain in the NAc were not dependent on the abstinence or relapse-like phase, indicating a specific change for CFA-treated female rats (**Figure 3B**). In fact, inflammatory pain induces microglial activation in both periods since its expression is significantly higher, about 20% higher, for CFA-females than for SAL-females in both abstinence (p = 0.001 and reintroduction (p = 0. 007).

Very interestingly, no significant changes were observed when comparing groups in male rats for neither PFC (**Figure 3B**), NAc (**Figure 3D**), nor VTA (**Figure 3F**).

#### 3.2.3 Downregulation and Upregulation of IL1β and IL10 in the NAc but Not in the PFC of CFA-Treated Female Rats Follow a Different Pattern Than That Observed in Saline-Treated Female Rats

Pro-inflammatory and anti-inflammatory cytokines mainly regulate the activity of several cells, above all immune cells, promoting cell communication. Since we observed alterations in microglial activation in PFC and NAc from female rats, we analyzed the levels of the pro-inflammatory cytokine IL1β and the anti-inflammatory one IL10 in those areas from females.

**Figure 4:**
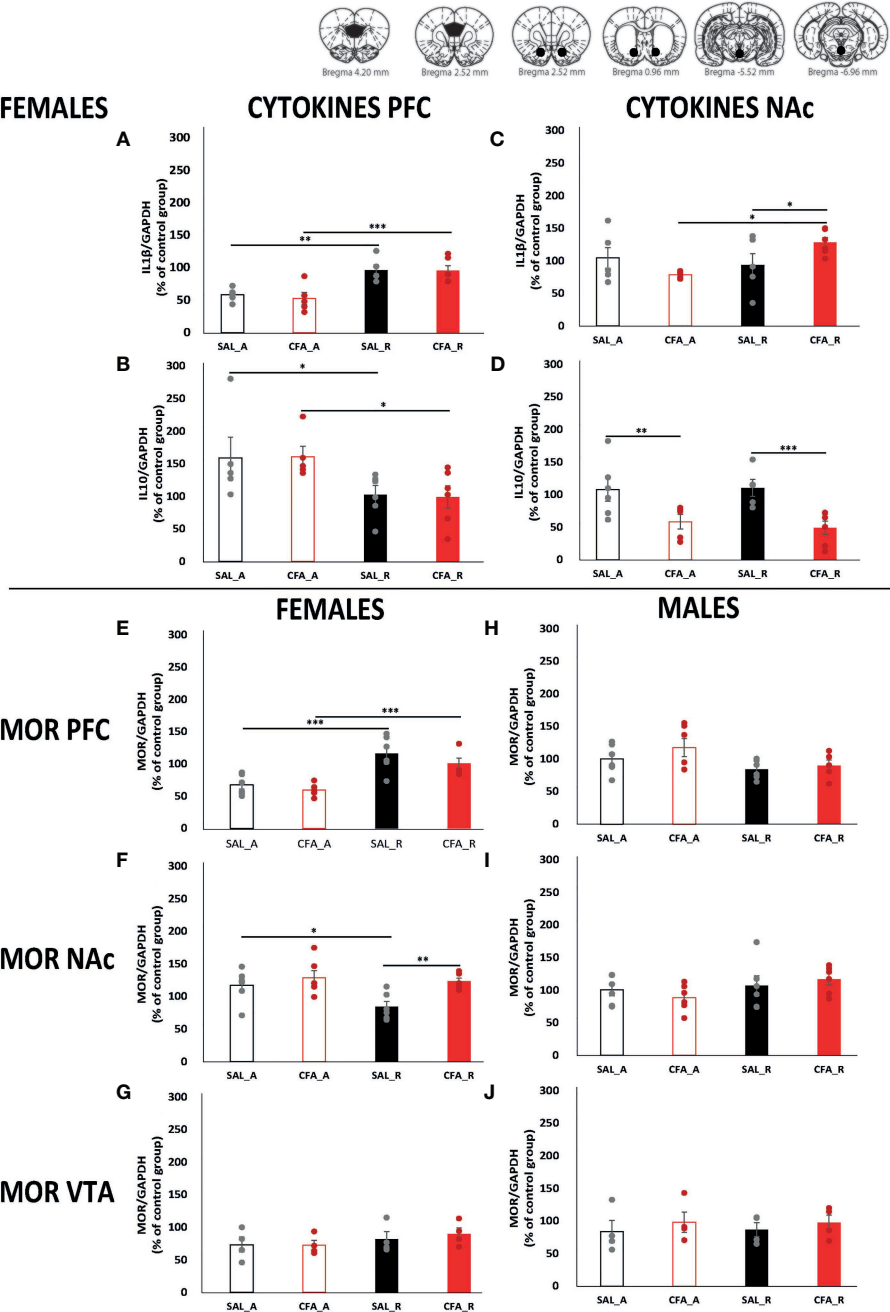
Alterations of inflammatory mediators during the abstinence and the alcohol reintroduction periods in the presence or absence of inflammatory pain in female and male rats. Data are expressed as mean ± SEM of protein levels in % relative to control (n = 4–6/group). Black and red bars represent saline and CFA-females, respectively, during abstinence (empty bars) and after abstinence (filled bars) periods. Points represent the individual data from each animal from the group. On top, schematic representations of the punched brain areas harvested to perform the western blots. Graphs **(A–G)** gather the protein analysis of female rats and graphs **(H–J)** those from male rats; PFC, NAc, and VTA protein analyses are represented in the following order, PFC: **(A, B, E, H)**; NAc: **(C, D, F, I)**; VTA: **(G, J)**. The proteins analyzed are IL1β **(A, C)**, IL10 **(B, D)**, and MOR **(E–J)**. Asterisks mark statistically significant differences in the Bonferroni multiple-comparison test applied when the two-way ANOVA detected significant differences in the main effects or in the interaction (*p < 0.05, **p < 0.01, ***p < 0.005). CFA, complete Freund adjuvant; SAL, saline; A, abstinence period; R, reintroduction period; PFC, prefrontal cortex; NAc, nucleus accumbens; VTA, ventral tegmental area; IL1b, interleukin 1b; IL10, interleukin 10; MOR, mu-opioid receptor.

On the one hand, regarding PFC, the levels of IL1β were significantly lower during abstinence than after reintroduction regardless of the presence of inflammatory pain (**Figure 4A**. Bonferroni multiple comparison: SAL_A *vs.* SAL_R, p = 0.001; CFA_A *vs.* CFA_R, p = 0.0001). Interestingly, we observed the opposite changes in IL10, indicating a different regulation of pro- and anti-inflammatory events. The levels of IL10 suffered a decrease after reintroduction of the alcohol beverages regardless of the presence of inflammatory pain (**Figure 4B**. Bonferroni multiple comparisons: SAL_A *vs.* SAL_R, p = 0.037; CFA_A *vs.* CFA_R, p = 0.025). On the other hand, in NAc of CFA-treated female rats, the levels of IL1β significantly increased after reintroduction (p = 0.006) and also when compared to SAL-animals (p = 0.045). Furthermore, when analyzing the levels of the anti-inflammatory cytokine IL10, the development of inflammatory pain significantly decreased the levels of IL10 during abstinence (0.011) and after reintroduction (p = 0.002) when compared to SAL-females (**Figure 4D**).

#### 3.2.4 Alcohol and Inflammatory Pain Impacts MOR Expression Patterns in PFC and NAc During Abstinence and Reintroduction Phases in Female Rats: Specific Effect of the Presence of Pain in the NAc

MOR activation during reintroduction phases in areas of the MSCL has shown to play a crucial role in alcohol-relapse-like behavior (41). To investigate dynamic changes of MOR expression in the MSCL areas of interest in the abstinence and reintroduction phases of male and female rats in our model, we measured its relative expression levels by Western blot. **Figure 4** shows MOR expression in saline and CFA-treated female rats during abstinence and after alcohol reintroduction, and in control females in the same conditions. The two-way ANOVA tests performed confirmed that MOR levels were altered depending on the alcohol drinking period, the brain area studied, and the sex.

No changes were observed in the MOR expression of male rats in PFC (**Figure 4H**), NAc (**Figure 4I**), and VTA (**Figure 4J**). Moreover, the expression of MOR in VTA from female rats did not show any significant alterations between groups (**Figure 4G**).

Interestingly, MOR levels were significantly lower during abstinence than after reintroduction regardless of the presence of inflammatory pain in PFC from female rats (**Figure 4E**, Bonferroni multiple comparisons: SAL_A *vs.* SAL_R, p = 0.0001; CFA_A *vs.* CFA_R, p = 0.001). Finally, very interesting data show alterations in the pattern of MOR expression depending on the presence of pain in the NAc (**Figure 4F**). In the case of saline-treated female rats, MOR expression was reduced after the reintroduction of the alcohol beverages when compared to the abstinence period (p = 0.018). In addition, inflammatory pain significantly increases the levels of MORs by 50% in female rats after reintroduction when compared to saline-treated female rats (p = 0.006).

## 4 Discussion

Our present results show dynamic alterations of microglial activation and neuroinflammatory mediator, cytokine, and MOR expression through the abstinence and reintroduction phases of a sex-dependent inflammatory pain-induced alcohol relapse rat model. Some of these alterations demonstrate to be dependent on the sex, abstinence, or reintroduction to alcohol drinking, the MCLS areas studied or their interaction providing new insights into neuroinflammatory properties of alcohol and its interaction with pain-induced alterations in these areas. Firstly, our drinking behavior results in female rats confirm that CFA-treated female rats show ADE, but the saline-treated ones do not as we have previously described in (25). Moreover, this group exhibited alterations in pNFκB and COX2, microglial activation (measured as IBA1 expression), and expression of IL1β and IL10 together with MOR in the PFC, NAc, and/or VTA, with those unique changes in NAc being of a special relevance. Indeed, these results showed a decrease of microglial activation in the PFC only during abstinence, and an augmentation of the microglial activation in the NAc of CFA-female rats in both abstinence and reintroduction phases. Additionally, the expression of pNFκB and IL1β also evidenced these dynamic changes through these two phases following similar expression patterns in both areas. As mentioned, these changes in NAc were observed in the presence of inflammatory pain only in female rats, which was the condition that triggered alcohol-relapse-like behavior in our animal model. Additionally, the expression of cytokine IL10 showed a different profile than the IL1β one, indicating anti-inflammatory processes occurring only during abstinence in the PFC of CFA-female rats, but not during the reintroduction phase in PFC or in the NAc. All in all, these data might indicate a downregulation of microglial activation and pro-inflammatory processes during abstinence in the PFC regardless of the presence of pain, whereas an upregulation can be observed in the NAc during abstinence that is maintained during the reintroduction phase only in CFA-treated females. Furthermore, we also investigated the expression of MORs in the same areas of the MCLS in the abstinence and reintroduction phases of our animal model. Notably, the same dynamics were also observed in the case of the MOR expression, suggesting that the already described interaction between MORs and neuroinflammation might also be underlying the adaptations developed during the abstinence and reintroduction phases.

To our knowledge, this is the first study analyzing neuroinflammatory mediators, microglial activation, and cytokines in the abstinence and alcohol relapse phases of a model that allows to compare sex-dependent behavior. Several reports have shown that chronic alcohol induces neuroinflammation, probably through TLR4, to produce release of diverse pro-inflammatory cytokines (21) and cause neural damage (22). These effects of alcohol exposure seem to be more intense in females than in males (42, 43), and, as shown in **Figure 2**, although the development of pain itself during abstinence altered some of these neuroinflammatory mediators in both male and female rats, our data failed to show increases in neuroinflammatory mediators in male rats. One plausible explanation might be related with the experimental protocol which allows free drinking, instead of forced alcohol intake shown in other studies (20, 44, 45), or the fact that sacrifices were carried out 24 h after the last alcohol consumption, allowing increased neuroinflammatory markers to return to normal levels. Very interestingly, the effect of inflammatory pain in neuroinflammation and microglial activation (see **Figures 2C, M, P**, **3C**) has recently captured the attention of researchers. Recent publications have shown that systemic inflammation leads also to the elevated presence of inflammatory mediators in the brain, which correlates with depressive-like behaviors in patients but also with negative affective state and alterations in neuronal excitability of neurons of NAc in mice (46–48). In line with these data, adaptations derived from the presence of pain might also account for the neuroinflammatory effects produced by alcohol, as is shown in our case for some studied proteins in CFA-treated female rats during abstinence and/or reintroduction phases.

Increasing evidence involves the innate immune system in alcohol drinking (49) and alcohol relapse behavior in female rats. Thus, in a very interesting set of studies, Ezquer and colleagues showed that administration of the secretome or exosomes from human mesenchymal cells prevented the increase in alcohol drinking after a period of abstinence in their female rat model (24, 50). In accordance with these results, our present data showed an increase of IBA1 staining in NAc of CFA-treated female rats during abstinence. Interestingly, in this same experimental group, IL10 expression in the NAc was downregulated, suggesting a pro-inflammatory state that might play a role in promoting ADE since we did not observe these changes in any of the non-relapsing groups. In addition to that, when alcohol was reintroduced, and presumably due to the presence of alcohol, the proinflammatory state was maintained as data of IBA1, pNFκB, and IL1β expression evidenced. Contrary to that, in PFC, CFA-treated female rats showed an anti-inflammatory state during abstinence that was reverted to pro-inflammatory state after alcohol reintroduction most likely as a consequence of alcohol intake and regardless of the presence of pain. In this case, IBA1 staining and the expression of pNFκB and IL1β were significantly lower, together with significantly higher levels of IL10 during abstinence. In line with these results, it has recently been shown that chronic intermittent access to ethanol and lipopolysaccharide exposure differentially alters PFC and NAc microglia soma volume 10 h after the end of the alcohol IA protocol, with microglia from PFC being more affected than that from NAc (51). These brain-region-dependent alterations might be a consequence of the presence of different subtypes of microglia populating in each area (52). It is also very interesting to observe that these alterations in the expression of IBA1 and IL1β in NAc of saline-treated females are different from those observed in CFA-treated females. Altogether, these significantly different alterations for CFA-treated female rats might be taken into consideration since they could potentially explain the inflammatory pain-induced alcohol relapse phenomenon that we observe in our model with only female rats.

Finally, abstinence and alcohol reintroduction did not increase microglial activation in VTA which is in accordance with previous results showing no effect of alcohol on microglial activation in VTA from postmortem human brains (53). From all the proteins studied, only pNFκB and iNOS after reintroduction of alcohol showed significant alterations in the VTA, but because of the involvement of this transcription factor in different physiological events, it is difficult to interpret its significance in the observed behavior. Moreover, we have not measured the levels of the non-phosphorylated form of NFκB, which is also a limitation of the study that makes even more difficult to interpret these results. Further studies should address the consequences of this increase in the pNFκB observed after alcohol reintroduction and its differences in saline versus CFA-treated females.

Interestingly, our results connect microglial activation and the expression of IL1β with MOR levels in relevant brain areas of MCLS. This correlation has already been described in a neonatal alcohol intake model in rats (12). In this report, the authors suggested that microglial MOR activation enhances the neuroinflammatory response, as other papers have also previously reported (11, 12, 54). In addition, the presence of neuroinflammation, and, more specifically, the increase of IL-1β, might influence MOR expression (13–16). In general, our results support these previous reports showing a bidirectional relationship between MORs and IL-1β. Nonetheless, it is interesting to mention that in the NAc, MOR expression remain unaltered during abstinence and after reintroduction to alcohol in CFA-female rats, whereas IL1β significantly increases only after the reintroduction of alcohol. Anyway, IL1β and MOR expressions are both significantly increased in CFA-treated females in the reintroduction phase (**Figures 4C, F**). This lack of correlation with IL1β in the abstinence period might be a consequence of different timelines in the expression patterns. In addition to this, MOR activation can also reduce the levels of anti-inflammatory cytokines such as IL10 (55, 56), and, as our results evidence, in NAc and PFC of females, MORs and IL10 expression levels are opposed along the different phases. The presence of neuroinflammation, and alterations of MCLS MOR expression, are suggested to underlie alcohol neurobiological effects and relapse-like behavior (23, 24, 57–60). All in all, these results point to neuroinflammation-MOR cross talk as a relevant piece in the abstinence and relapse neurobiological substrate puzzle.

Collectively, our results suggest that microglial activation and the resulting neuroinflammation, together with MOR level alterations in PFC and NAc, are likely to participate in an inflammatory pain-induced relapse-like behavior in female rats. Nonetheless, further research to clarify the role of the glial-neuron cross talk in alcohol relapse is warranted.

## Data Availability Statement

The raw data supporting the conclusions of this article will be made available by the authors, without undue reservation.

## Ethics Statement

The animal study was reviewed and approved by the Ethics Committee in Experimentation and Animal Welfare of the University of Valencia and the local Goverment of Valencia (Conselleria d’Agricultura, Desenvolupament Rural, Emergéncia Climática i Transició Ecológica).

## Author Contributions

Conceptualization, LH and JC. Methodology, JC, JDL, YC-J, and LH. Formal analysis, JC and LH. Investigation, JC, JDL, and YC-J. Writing—original draft, JC. Resources, LH. Supervision, AP and LH. Writing—review and editing, JC, JDL, YC-J, AP, and LH. All authors contributed to the article and approved the submitted version.

## Funding

This study has been supported by Spanish Ministerio de Ciencia e Innovación PID2019-109823RB-I00 (LH) and by Spanish Ministerio de Sanidad, Delegación del Gobierno para el Plan Nacional sobre Drogas PNSD2019I038 (LH). JC is supported by a Atracció de Talent PhD Fellowship from the University of Valencia (UV-INV-PREDOC-1327981).

## Conflict of Interest

The authors declare that the research was conducted in the absence of any commercial or financial relationships that could be construed as a potential conflict of interest.

## Publisher’s Note

All claims expressed in this article are solely those of the authors and do not necessarily represent those of their affiliated organizations, or those of the publisher, the editors and the reviewers. Any product that may be evaluated in this article, or claim that may be made by its manufacturer, is not guaranteed or endorsed by the publisher.

## Supplementary Material

Supplementary Figure 1 Representative images of DAPI and IBA1 staining from each group in females taken with 20x objective. White scale bars represent 100 μm. Brain schematics represent the areas where the pictures were taken (Paxinos and Watson 2006). CFA, complete Freund Adjuvant; SAL, saline; C, control; A, abstinence period; R, re-introduction period; PFC, prefrontal cortex; NAc, nucleus accumbens; VTA, ventral tegmental area; IBA1, ionized calcium-binding adapter molecule 1; DAPI, 4′,6-diamidino-2-phenylindole.

Supplementary Figure 2 Representative images of DAPI and IBA1staining from each group in males taken with 20x objective. White scale bars represent 100 μm. Brain schematics represent the areas where the pictures were taken (Paxinos and Watson 2006). CFA, Complete Freund Adjuvant; SAL, saline; C, control; A, abstinence period; R, re-introduction period; PFC, prefrontal cortex; NAc, nucleus accumbens; VTA, ventral tegmental area; IBA1, ionized calcium-binding adapter molecule 1; DAPI, 4′,6-diamidino-2-phenylindole.

Supplementary Figure 3 Representative measured bands obtained in the western blot from each group and each analysed protein. CFA, Complete Freund Adjuvant; C, control; SAL, saline; A, abstinence period; R, reintroductionperiod; PFC, prefrontal cortex; NAc, nucleus accumbens; VTA, ventral tegmental area; pNF-kB, phosphorylated Nuclear Factor kB; iNOS, inducible Nitric Oxide Synthase; COX2, Cyclooxygenase 2; IL1b, Interleukin 1b; IL10, Interleukin 10; MOR, Mu-Opioid Receptor.

Supplementary Table 1 Statistical analysis for **Figures 2**–**4** (Two-Way ANOVA, SPSS 26) Partial Eta2: proportion of explained variance, prefrontal cortex (PFC), nucleus accumbens (NAc), ventral tegmental area (VTA), phosphorylated Nuclear Factor κB (pNF-κB), inducible Nitric Oxide Synthase (iNOS), Cyclooxygenase 2 (COX2), Interleukin 1β (IL1β), Interleukin 10 (IL10), Mu Opioid Receptor (MOR) and ionized calcium-binding adapter molecule 1 (IBA1).

Supplementary Table 2 Summary of the significant changes observed in the immunofluorescence and western blot analysis. In yellow we present significant effects in main variable abstinence and re-introduction periods, in blue significant effects in main effect saline-treated and CFA-treated rats, in green the significant effects for both and in red no differences. + and - simbols are used to indicate if the group presents higher (+) or lower (-) levels of the protein of analysis when compared to another group with the Bonferroni multiple comparisons. In green cell two simbols are provided, the first one to indicate differences between abstinence and re-introduction and the second one to indicate differences between saline- and CFA-treated rats. CFA, Complete Freund Adjuvant; SAL, saline; A, abstinence period; R, re-introduction period; PFC, prefrontal cortex; NAc, nucleus accumbens.
