# Supplementary figures and images for: Neuroimmune and Mu-Opioid Receptor Alterations in the Mesocorticolimbic System in a Sex-Dependent Inflammatory Pain-Induced Alcohol Relapse-Like Rat Model
Source: Front Immunol. 2021 Sep 20;12:689453. doi: 10.3389/fimmu.2021.689453 (PMC8488159; doi:10.3389/fimmu.2021.689453)

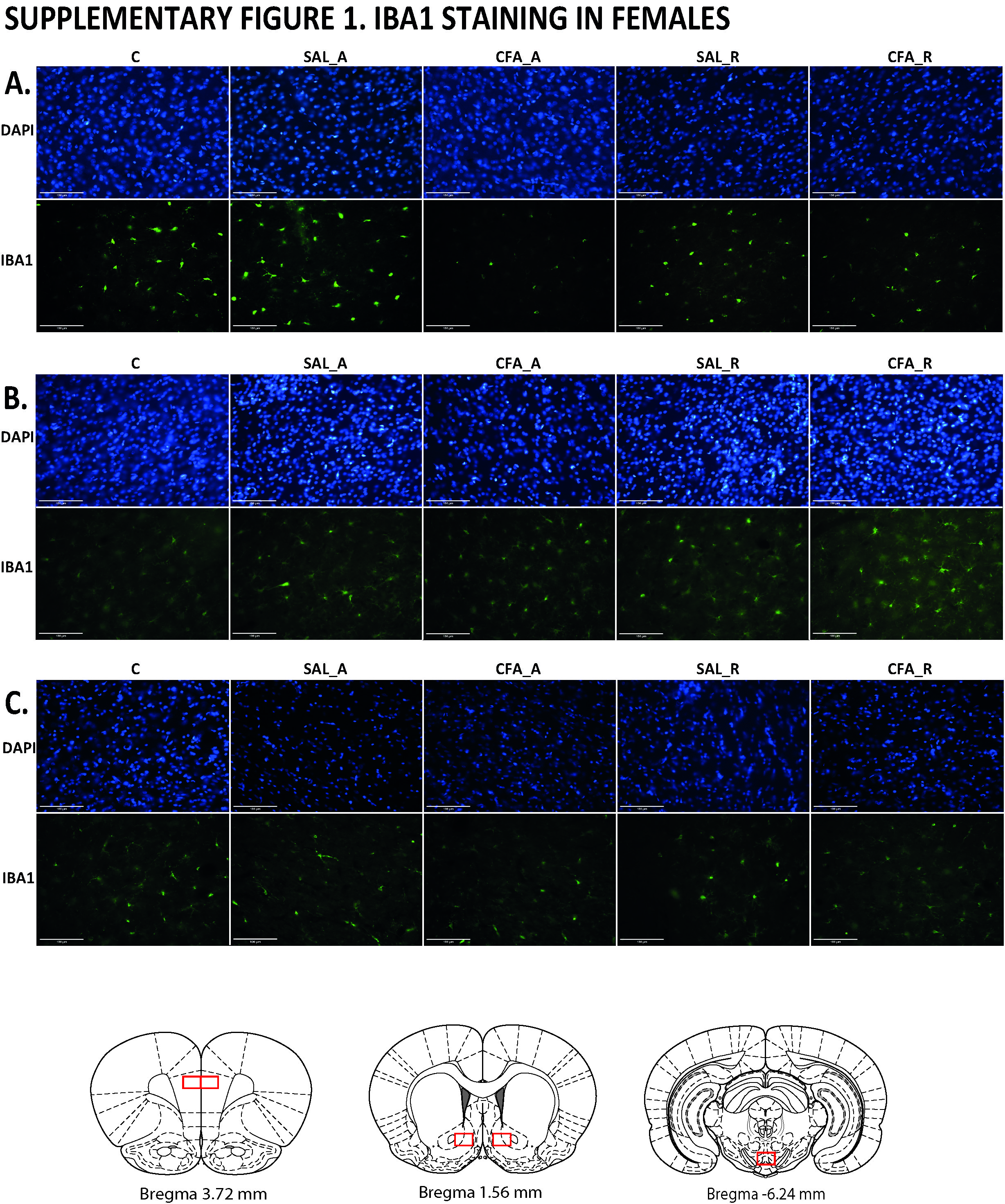

Supplement: Supplementary Figure 1 — Representative images of DAPI and IBA1 staining from each group in females taken with 20x objective. White scale bars represent 100 μm. Brain schematics represent the areas where the pictures were taken (Paxinos and Watson 2006). CFA, complete Freund Adjuvant; SAL, saline; C, control; A, abstinence period; R, re-introduction period; PFC, prefrontal cortex; NAc, nucleus accumbens; VTA, ventral tegmental area; IBA1, ionized calcium-binding adapter molecule 1; DAPI, 4′,6-diamidino-2-phenylindole. [file Image_1.jpeg]

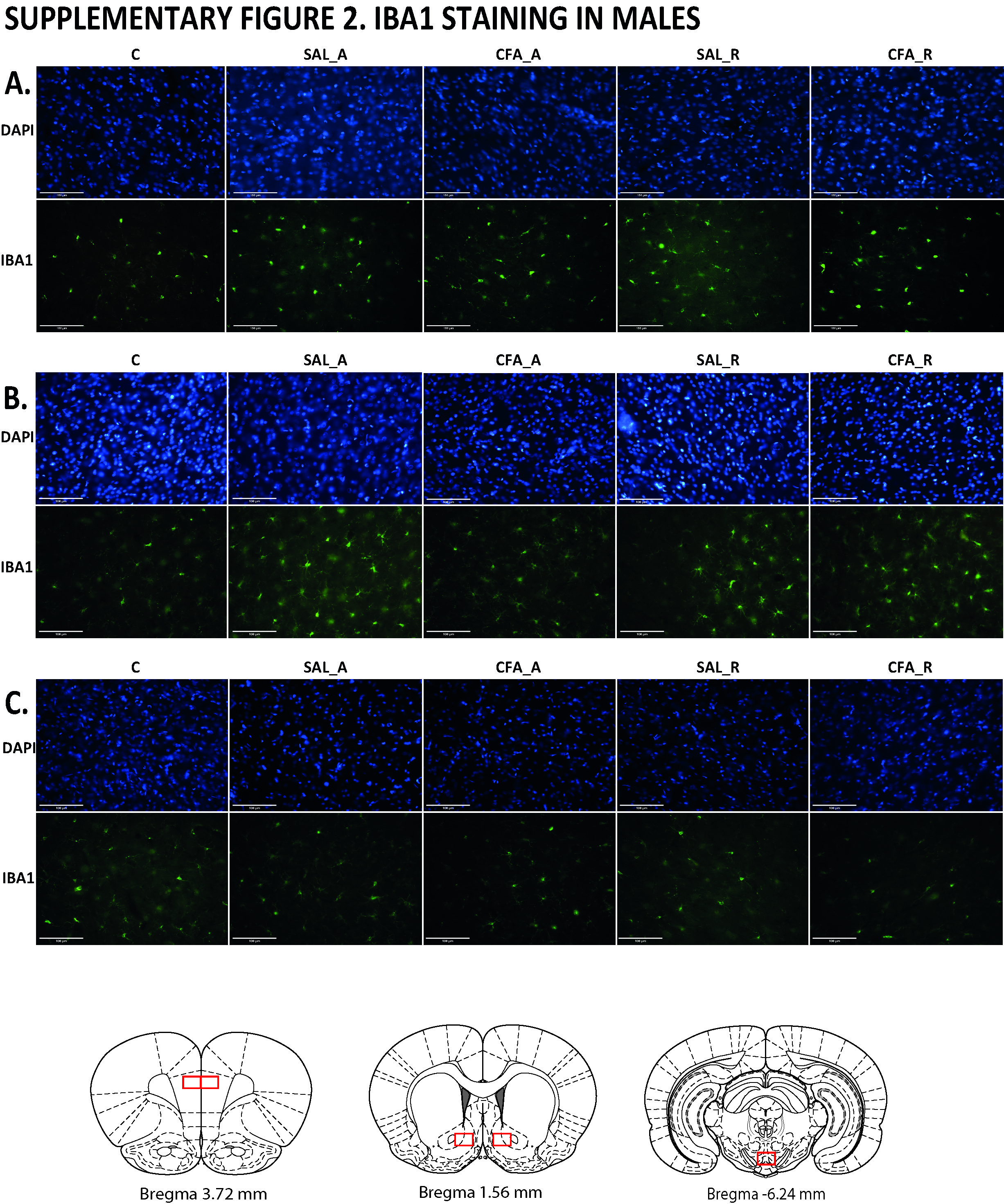

Supplement: Supplementary Figure 2 — Representative images of DAPI and IBA1staining from each group in males taken with 20x objective. White scale bars represent 100 μm. Brain schematics represent the areas where the pictures were taken (Paxinos and Watson 2006). CFA, Complete Freund Adjuvant; SAL, saline; C, control; A, abstinence period; R, re-introduction period; PFC, prefrontal cortex; NAc, nucleus accumbens; VTA, ventral tegmental area; IBA1, ionized calcium-binding adapter molecule 1; DAPI, 4′,6-diamidino-2-phenylindole. [file Image_2.jpeg]

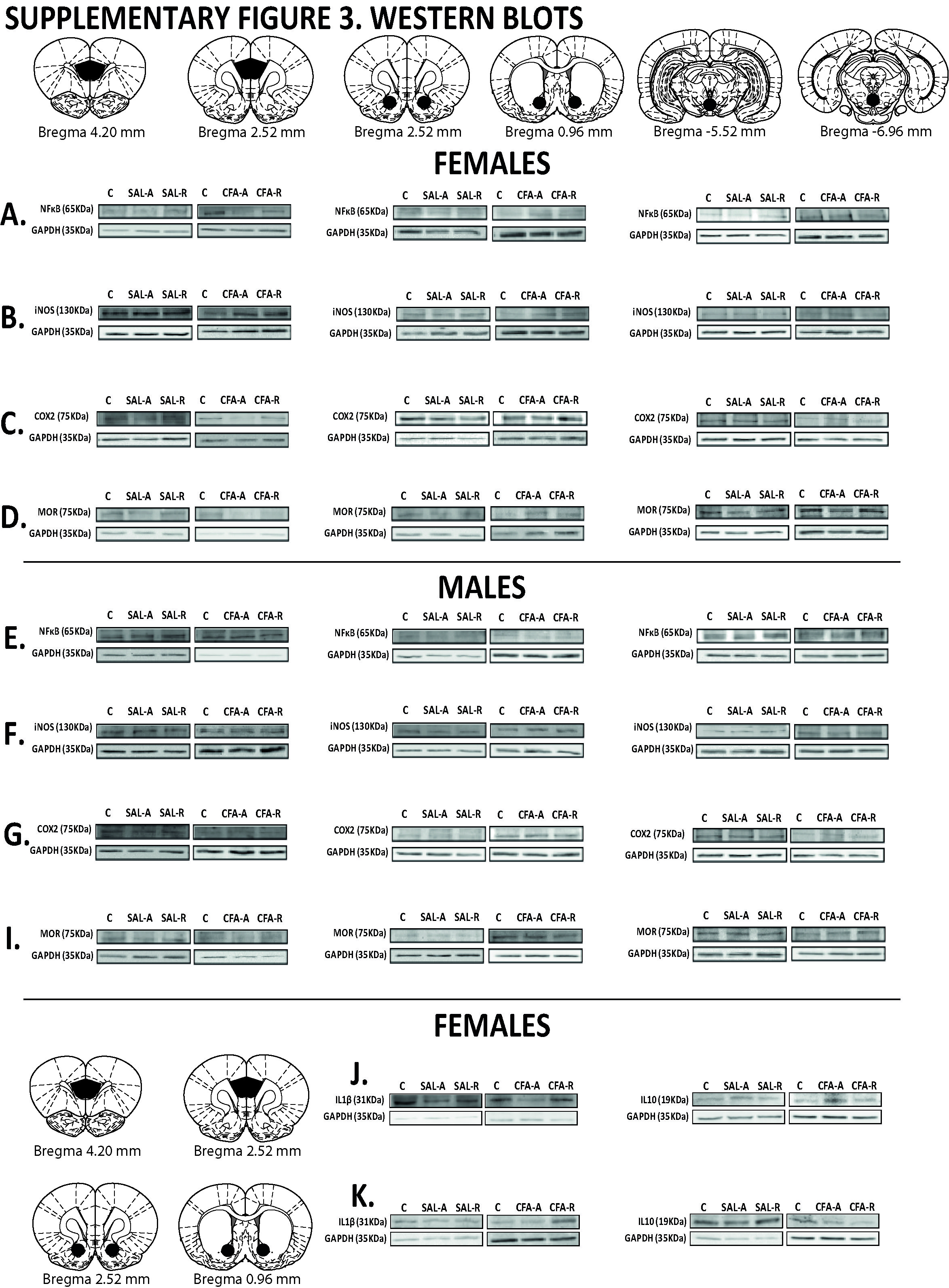

Supplement: Supplementary Figure 3 — Representative measured bands obtained in the western blot from each group and each analysed protein. CFA, Complete Freund Adjuvant; C, control; SAL, saline; A, abstinence period; R, reintroductionperiod; PFC, prefrontal cortex; NAc, nucleus accumbens; VTA, ventral tegmental area; pNF-kB, phosphorylated Nuclear Factor kB; iNOS, inducible Nitric Oxide Synthase; COX2, Cyclooxygenase 2; IL1b, Interleukin 1b; IL10, Interleukin 10; MOR, Mu-Opioid Receptor. [file Image_3.jpeg]
