# Supplementary material for: Neuroimmune and Mu-Opioid Receptor Alterations in the Mesocorticolimbic System in a Sex-Dependent Inflammatory Pain-Induced Alcohol Relapse-Like Rat Model
Source: Front Immunol. 2021 Sep 20;12:689453. doi: 10.3389/fimmu.2021.689453 (PMC8488159; doi:10.3389/fimmu.2021.689453)
Supplement: Supplementary Table 1 — Statistical analysis for Figures 2–4 (Two-Way ANOVA, SPSS 26) Partial Eta2: proportion of explained variance, prefrontal cortex (PFC), nucleus accumbens (NAc), ventral tegmental area (VTA), phosphorylated Nuclear Factor κB (pNF-κB), inducible Nitric Oxide Synthase (iNOS), Cyclooxygenase 2 (COX2), Interleukin 1β (IL1β), Interleukin 10 (IL10), Mu Opioid Receptor (MOR) and ionized calcium-binding adapter molecule 1 (IBA1). [file Table_1.pdf]

Figure number	Statistical Test	Factor name	F-value	p-value	Partial Eta^2
Figure 2A pNFκB PFC females	Two-Way ANOVA	**Abstinence**	F(1,24) = 19.189	<0.001*	0.49
		**Pain**	F(1,24) = 0.120	0.732	0.006
		**Intersection**	F(1,24) = 1.165	0.293	0.055
Figure 2B pNFκB NAc females	Two-Way ANOVA	**Abstinence**	F(1,24) = 2.650	0.119	0.117
		**Pain**	F(1,24) = 2.476	0.131	0.110
		**Intersection**	F(1,24) = 0.222	0.643	0.011
Figure 2C pNFκB VTA females	Two-Way ANOVA	**Abstinence**	F(1,16) = 2.573	0.135	0.177
		**Pain**	F(1,16) = 13.311	0.003*	0.526
		**Intersection**	F(1,16) = 38.055	<0.001*	0.760
Figure 2D iNOS PFC females	Two-Way ANOVA	**Abstinence**	F(1,24) = 12.187	0.002*	0.379
		**Pain**	F(1,24) = 1.200	0.286	0.057
		**Intersection**	F(1,24) = 0.099	0.756	0.005
Figure 2E iNOS NAc females	Two-Way ANOVA	**Abstinence**	F(1,24) = 0.108	0.745	0.005
		**Pain**	F(1,24) = 2.043	0.168	0.093
		**Intersection**	F(1,24) = 0.663	0.425	0.032
Figure 2F iNOS VTA females	Two-Way ANOVA	**Abstinence**	F(1,16) = 0.743	0.406	0.058
		**Pain**	F(1,16) = 7.612	0.017*	0.388
		**Intersection**	F(1,16) = 1.489	0.246	0.110
Figure 2G COX2 PFC females	Two-Way ANOVA	**Abstinence**	F(1,24) = 15.714	<0.001*	0.440
		**Pain**	F(1,24) = 0.100	0.755	0.005
		**Intersection**	F(1,24) = 5.614	0.028*	0.219
Figure 2H COX2 NAc females	Two-Way ANOVA	**Abstinence**	F(1,24) = 1.003	0.319	0.048
		**Pain**	F(1,24) = 2.467	0.132	0.110
		**Intersection**	F(1,24) = 1.748	0.201	0.080
Figure 2I COX2 VTA females	Two-Way ANOVA	**Abstinence**	F(1,16) = 0.909	0.359	0.070
		**Pain**	F(1,16) = 0.857	0.373	0.067
		**Intersection**	F(1,16) = 0.743	0.405	0.058
Figure 2J pNFκB PFC males	Two-Way ANOVA	**Abstinence**	F(1,24) = 3.751	0.067	0.158
		**Pain**	F(1,24) = 3.033	0.097	0.132
		**Intersection**	F(1,24) = 2.722	0.115	0.120
Figure 2K pNFκB NAc males	Two-Way ANOVA	**Abstinence**	F(1,24) = 0.494	0.490	0.024
		**Pain**	F(1,24) = 0.016	0.902	0.001
		**Intersection**	F(1,24) = 0.104	0.751	0.005

Figure 2L<br>pNFκB VTA<br>males	Two-Way ANOVA	**Abstinence**	F(1,16) = 2.932	0.113	0.196
		**Pain**	F(1,16) = 0.515	0.487	0.041
		**Intersection**	F(1,16) = 0.021	0.888	0.002
Figure 2M<br>iNOS PFC<br>males	Two-Way ANOVA	**Abstinence**	F(1,24) = 12.758	0.002*	0.389
		**Pain**	F(1,24) = 16.799	0.001*	0.457
		**Intersection**	F(1,24) = 10.083	0.005*	0.335
Figure 2N<br>iNOS NAc<br>males	Two-Way ANOVA	**Abstinence**	F(1,24) = 0.112	0.742	0.006
		**Pain**	F(1,24) = 2.967	0.100	0.129
		**Intersection**	F(1,24) = 0.839	0.371	0.040
Figure 2O<br>iNOS VTA<br>males	Two-Way ANOVA	**Abstinence**	F(1,16) = 1.457	0.251	0.108
		**Pain**	F(1,16) = 0.219	0.648	0.018
		**Intersection**	F(1,16) = 0.471	0.505	0.038
Figure 2P<br>COX2 PFC<br>males	Two-Way ANOVA	**Abstinence**	F(1,24) = 4.426	0.048*	0.181
		**Pain**	F(1,24) = 6.663	0.018*	0.250
		**Intersection**	F(1,24) = 3.523	0.075	0.150
Figure 2Q<br>COX2 NAc<br>males	Two-Way ANOVA	**Abstinence**	F(1,24) = 1.003	0.329	0.048
		**Pain**	F(1,24) = 2.467	0.132	0.110
		**Intersection**	F(1,24) = 1.748	0.201	0.080
Figure 2R<br>COX2 VTA<br>males	Two-Way ANOVA	**Abstinence**	F(1,16) = 0.918	0.357	0.071
		**Pain**	F(1,16) = 0.538	0.477	0.043
		**Intersection**	F(1,16) = 0.150	0.709	0.012
Figure 3A<br>IBA1 PFC<br>females	Two-Way ANOVA	**Abstinence**	F(1,24) = 3.948	0.058	0.141
		**Pain**	F(1,24) = 2.058	0.165	0.079
		**Intersection**	F(1,24) = 6.550	0.017*	0.214
Figure 3B<br>IBA1 PFC<br>males	Two-Way ANOVA	**Abstinence**	F(1,24) = 0.067	0.800	0.006
		**Pain**	F(1,24) = 0.014	0.908	0.001
		**Intersection**	F(1,24) = 0.134	0.721	0.011
Figure 3C<br>IBA1 NAc<br>females	Two-Way ANOVA	**Abstinence**	F(1,24) = 0.091	0.765	0.004
		**Pain**	F(1,24) = 24.114	<0.001*	0.501
		**Intersection**	F(1,24) = 0.537	0.471	0.0022
Figure 3D<br>IBA1 NAc<br>males	Two-Way ANOVA	**Abstinence**	F(1,24) = 0.039	0.847	0.003
		**Pain**	F(1,24) = 1.202	0.294	0.091
		**Intersection**	F(1,24) = 1.043	0.327	0.080
Figure 3E		**Abstinence**	F(1,24) = 2.019	0.168	0.078

IBA1 VTA	Two-Way ANOVA	**Pain**	F(1,24) = 3.168	0.088	0.117
females		**Intersection**	F(1,24) = 0.555	0.463	0.023
Figure 3F		**Abstinence**	F(1,24) = 1.297	0.277	0.098
IBA1 VTA	Two-Way ANOVA	**Pain**	F(1,24) = 1.208	0.293	0.091
males		**Intersection**	F(1,24) = 1.382	0.263	0.103
Figure 4A		**Abstinence**	F(1,24) = 37.202	<0.001*	0.650
IL1β PFC	Two-Way ANOVA	**Pain**	F(1,24) = 0.219	0.645	0.011
females		**Intersection**	F(1,24) = 0.142	0.710	0.007
Figure 4B		**Abstinence**	F(1,24) = 10.896	0.004*	0.353
IL10 PFC	Two-Way ANOVA	**Pain**	F(1,24) = 0.001	0.977	0.001
females		**Intersection**	F(1,24) = 0.017	0.896	0.001
Figure 4C		**Abstinence**	F(1,24) = 3.004	0.098	0.131
IL1β NAc	Two-Way ANOVA	**Pain**	F(1,24) = 0.142	0.710	0.007
females		**Intersection**	F(1,24) = 7.044	0.015*	0.260
Figure 4D		**Abstinence**	F(1,24) = 0.072	0.792	0.007
IL10 NAc	Two-Way ANOVA	**Pain**	F(1,24) = 19.719	<0.001*	0.496
females		**Intersection**	F(1,24) = 0.231	0.636	0.011
Figure 4E		**Abstinence**	F(1,24) = 32.690	<0.001*	0.620
MOR PFC	Two-Way ANOVA	**Pain**	F(1,24) = 2.223	0.152	0.100
females		**Intersection**	F(1,24) = 0.210	0.651	0.010
Figure 4F		**Abstinence**	F(1,24) = 4.492	0.047*	0.183
MOR Nac	Two-Way ANOVA	**Pain**	F(1,24) = 7.823	0.011*	0.281
females		**Intersection**	F(1,24) = 2.314	0.144	0.104
Figure 4G		**Abstinence**	F(1,16) = 1.668	0.221	0.122
MOR VTA	Two-Way ANOVA	**Pain**	F(1,16) = 0.133	0.721	0.011
females		**Intersection**	F(1,16) = 0.209	0.656	0.017
Figure 4H		**Abstinence**	F(1,24) = 5.345	0.032	0.211
MOR PFC	Two-Way ANOVA	**Pain**	F(1,24) = 1.493	0.236	0.069
males		**Intersection**	F(1,24) = 0.363	0.554	0.018
Figure 4I		**Abstinence**	F(1,24) = 2.596	0.123	0.115
MOR Nac	Two-Way ANOVA	**Pain**	F(1,24) = 0.008	0.930	0.001
males		**Intersection**	F(1,24) = 1.042	0.320	0.050
Figure 4J		**Abstinence**	F(1,16) = 0.005	0.944	0.001
MOR VTA	Two-Way ANOVA	**Pain**	F(1,16) = 0.807	0.387	0.063

males	**Intersection**	F(1,16) = 0.017	0.898	0.001
