# Supplementary material for: Neuroimmune and Mu-Opioid Receptor Alterations in the Mesocorticolimbic System in a Sex-Dependent Inflammatory Pain-Induced Alcohol Relapse-Like Rat Model
Source: Front Immunol. 2021 Sep 20;12:689453. doi: 10.3389/fimmu.2021.689453 (PMC8488159; doi:10.3389/fimmu.2021.689453)
Supplement: Supplementary Table 2 — Summary of the significant changes observed in the immunofluorescence and western blot analysis. In yellow we present significant effects in main variable abstinence and re-introduction periods, in blue significant effects in main effect saline-treated and CFA-treated rats, in green the significant effects for both and in red no differences. + and - simbols are used to indicate if the group presents higher (+) or lower (-) levels of the protein of analysis when compared to another group with the Bonferroni multiple comparisons. In green cell two simbols are provided, the first one to indicate differences between abstinence and re-introduction and the second one to indicate differences between saline- and CFA-treated rats. CFA, Complete Freund Adjuvant; SAL, saline; A, abstinence period; R, re-introduction period; PFC, prefrontal cortex; NAc, nucleus accumbens. [file Table_2.pdf]

Brain area	Protein	Sex	Figure	SAL_A	CFA_A	SAL_R	CFA_R
PFC	pNFκB	females	Figure 2A	-	-	+	+
		males	Figure 2J				
	iNOS	females	Figure 2D	+	+	-	-
		males	Figure 2M	-	+/+		-
	COX2	females	Figure 2G		-		+
		males	Figure 2P	-	+/+		-
	IBA1	females	Figure 3A	+	-/-		+
		males	Figure 3B				
	IL1β	females	Figure 4A	-	-	+	+
	IL10	females	Figure 4B	+	+	-	-
	MOR	females	Figure 4E	-	-	+	+
		males	Figure 4H				
NAc	pNFκB	females	Figure 2B				
		males	Figure 2K				
	iNOS	females	Figure 2E				
		males	Figure 2N				
	COX2	females	Figure 2H				
		males	Figure 2Q				
	IBA1	females	Figure 3C	-	+	-	+
		males	Figure 3D				
	IL1β	females	Figure 4C		-	-	+/+
	IL10	females	Figure 4D	+	-	+	-
	MOR	females	Figure 4F	+		-/-	+
		males	Figure 4I				
VTA	pNFκB	females	Figure 2C	-/-	+/+	+	-
		males	Figure 2L				
	iNOS	females	Figure 2F			-	+
		males	Figure 2O				
	COX2	females	Figure 2I				
		males	Figure 2R				
	IBA1	females	Figure 3E				
		males	Figure 3F				
	MOR	females	Figure 4G				
		males	Figure 4J				
